# Toxic epidermal necrolysis with thrombocytopenia induced by intravenous immunoglobulin: a case report and mini review

**DOI:** 10.1186/s40780-024-00405-2

**Published:** 2025-01-28

**Authors:** Yoshihiro Nishita, Masatoshi Taga, Nozomi Arakawa, Tomoki Ishida, Sawako Ochiai, Hiroto Ono, Fumiaki Taga, Togen Masauji

**Affiliations:** 1 Department of Pharmacy, Kanazawa Medical University Hospital, 1-1 Daigaku, Uchinada-Cho, Kahoku-Gun, Ishikawa, 920-0293 Japan; 2 Department of Dermatology, Kanazawa Medical University, 1-1 Daigaku, Uchinada-Cho, Kahoku-Gun, Ishikawa, 920-0293 Japan

**Keywords:** Drug eruptions, Immunoglobulin, Stevens–Johnson syndrome, Toxic epidermal necrolysis, Thrombocytopenia, Drug Monitoring

## Abstract

**Background:**

Toxic epidermal necrolysis (TEN), a severe cutaneous hypersensitivity reaction induced particularly by drugs, is diagnosed when there is a fever of ≥ 38 °C, mucocutaneous symptoms, a rash with multiple erythema, and skin peeling of ≥ 30% of the body surface area. The mortality rate of TEN is high, and thrombocytopenia during treatment can lead to severe outcomes. Intravenous immunoglobulin (IVIg) is used when steroids are ineffective in TEN and may improve mortality; however, thrombocytopenia is a rare adverse event associated with IVIg use. We report the case of thrombocytopenia during IVIg therapy for TEN. We also reviewed previous reports to learn more about the clinical course and mechanism of IVIg-induced thrombocytopenia.

**Case presentation:**

An 83-year-old man with end-stage renal failure on hemodialysis was diagnosed with TEN. After an inadequate response to pulse methylprednisolone therapy, IVIg (400 mg/kg/day) was administered for 5 days. He developed thrombocytopenia after IVIg administration, leading to the diagnosis of thrombocytopenia due to IVIg after excluding other diseases. The platelet count began to increase approximately 10 days after IVIg administration.

**Conclusions:**

When IVIg is administered for TEN, the risk of thrombocytopenia should be recognized and the platelet count should be carefully monitored.

## Background

Toxic epidermal necrolysis (TEN), a severe cutaneous hypersensitivity reaction induced particularly by drugs, is diagnosed when there is a fever of ≥ 38 °C, mucocutaneous symptoms, a rash with multiple erythema, and skin peeling of ≥ 30% of the body surface area. The mortality rate reportedly ranged from 15 to 33% for TEN [1–3], with even higher rates reported for older patients and those with renal failure [4].

Intravenous immunoglobulin (IVIg) is used when steroids are ineffective in TEN and has been reported to improve mortality [2]. However, thrombocytopenia is a rare adverse event of IVIg administration. Although thrombocytopenia has been reported in patients with bullous pemphigoid [5], no reports on IVIg-induced thrombocytopenia in TEN have been found in our literature review. Here, we report a case of thrombocytopenia during IVIg administration for TEN. We also reviewed previous reports to learn more about the clinical course and mechanism of IVIg-induced thrombocytopenia.

## Case presentation

An 83-year-old man with end-stage renal failure on hemodialysis and primary malignant lymphoma of the central nervous system was receiving tirabrutinib. On day 1, the patient was seen at the hospital with generalized erythema, which appeared three days earlier. A novel coronavirus antigen test was performed, and it showed positive findings. The patient was hospitalized for tirabrutinib or a novel coronavirus-associated generalized rash. Physical examination revealed mild hyperemia of the ocular mucosa, infiltrated and shiny lips with no blood crusts, pale erythema around the mouth, and dark-purple edematous erythema on the trunk. There were several targeted and flaccid blister lesions on the upper extremities, and erythema was noted. The rash extended to the central half of the thigh.

**Fig. 1:**
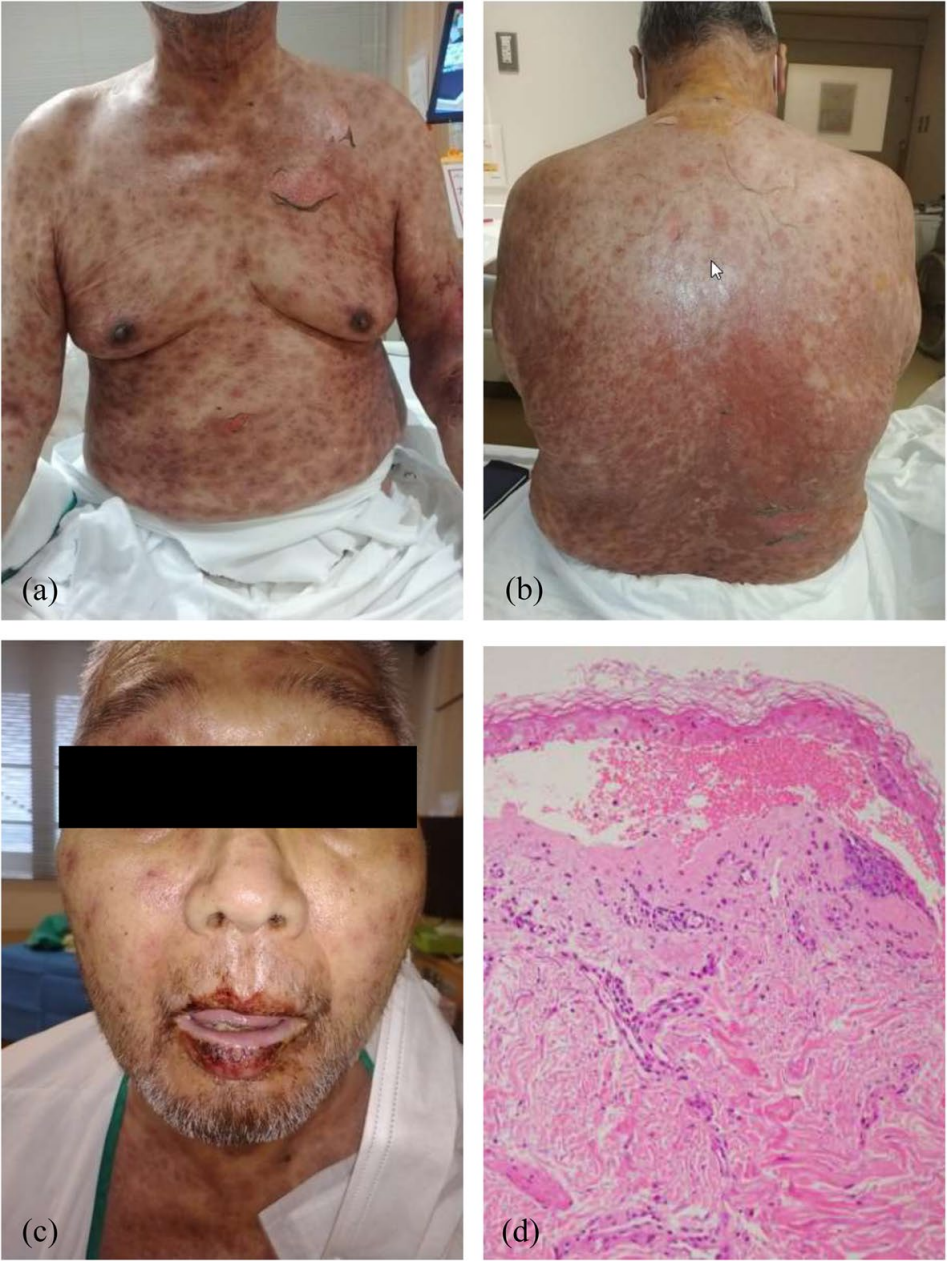
Clinical and pathological manifestations of this case. **a**, **b** Diffuse erythema with some erosion and flaccid blisters is observed on the trunk. **c** Eschar adhering to the lips. **d** Pathological findings showing extensive degenerative necrosis of the epidermis and edematous dermal epithelium (original magnification: × 100)

After admission, tirabrutinib and trimethoprim/sulfamethoxazole, which were listed as suspected drugs for skin rash, were discontinued. Remdecivir was administered to prevent the development of severe coronavirus infection. On day 3, mucocutaneous symptoms appeared and the epidermal necrosis area was 2%, leading to the diagnosis of Stevens–Johnson syndrome (SJS). Pulse methylprednisolone therapy (1 g/day) was administered for 3 days (Days 3–5). Although the coronavirus infection resolved without serious complications, the extent of epidermal necrosis continued to expand even after pulse methylprednisolone therapy, and the patient was diagnosed with TEN on day 7 (Fig. 1a, b, c). The pathological findings of the neck skin specimen submitted on day 7 revealed extensive degenerative necrosis of the epidermis, peeling, subepidermal blister formation, and mild perivasculitis, mainly in the edematous dermal epithelium with few eosinophils and lymphocytes (Fig. 1d). He was diagnosed with TEN.

**Fig. 2:**
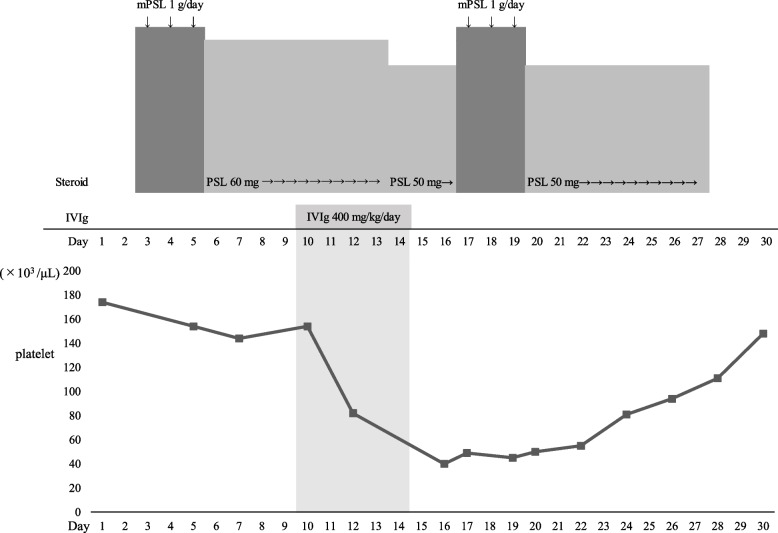
Course of treatment and platelet count changes. *PSL*, prednisolone; *mPSL*, methylprednisolone; *IVIg*, intravenous immunoglobulin

On day 10, IVIg (400 mg/kg/day) was administered for 5 days (Days 10–14). His platelet count decreased after the initiation of IVIg and further decreased from 154 × 10^3^ /μL on day 10 to 40 × 10^3^/μL on day 16. We ruled out ethylenediaminetetra acetic acid-dependent pseudothrombocytopenia as a cause of thrombocytopenia because the peripheral hemogram revealed no platelet aggregation. The rapid decline in platelet counts suggested thrombocytopenia might result from platelet consumption or destruction. Disseminated intravascular coagulation (DIC), antiphospholipid antibody syndrome (APS), and heparin-induced thrombocytopenia (HIT) were suspected as the causes of thrombocytopenia. FDP and PT-INR levels on day 16 were 3.9 μg/dL and 1.08, respectively, and antithrombin activity was only slightly decreased to 74.9%, indicating the absence of DIC. Given that the lupus anticoagulant level was 1.0 and the anticardiolipin beta2-glycoprotein I complex antibody on day 16 was below the detection sensitivity, APS was also ruled out. The anti-platelet factor 4/heparin antibody level was 1.1 U/mL; however, heparin was continued because there were no new thromboembolisms or microthrombi in the subcutaneous vessels on the pathological findings of the skin biopsy. Systemic lupus erythematosus (SLE) and ANCA-associated vasculitis were considered as possibilities. However, SLE was eliminated due to negative antinuclear antibodies; lack of muscle, joint, and neurological symptoms; and absence of hemolytic anemia. Similarly, ANCA-associated vasculitis was ruled out based on negative PR3-ANCA and MPO-ANCA test results. The platelet-associated IgG (PAIgG) level was high at 154 ng/10^7^ cells (Day 16). Given that there was partial improvement and worsening of symptoms after IVIg therapy, pulse methylprednisolone therapy (1 g/day) was again administered for 3 days (Days 17–19). The platelet count gradually increased, reaching 111 × 10^3^ /μL on day 28. Figure 2 shows the treatment course and platelet counts. The red and white blood cell counts did not decrease rapidly during the treatment period.

Although the drugs used during decreased platelet count were continued, except for IVIg, the platelet count did not decrease again, leading to the diagnosis of thrombocytopenia due to IVIg. The skin and mucous membrane findings gradually improved, and the patient was transferred to the general ward on day 49.

## Discussion and conclusions

In this paper, we report a case of thrombocytopenia induced by IVIg for TEN. To the best of our knowledge, this is the rare report of IVIg-induced thrombocytopenia in TEN. In this case, there were no clinical findings indicating acute TEN exacerbation, and the laboratory findings and clinical status were negative for myelosuppression and APS. We also suspected DIC, but this was also ruled out because no laboratory findings suggested the presence of increased microthrombosis, and the patient did not meet the diagnostic criteria for DIC according to the Japanese Society on Thrombosis and Hemostasis [6]. Although the anti-platelet factor 4/heparin antibodies were positive, the immunoassay using latex agglutination in this study may give false-positive results [7, 8]. HIT was also ruled out by the absence of thromboembolism or microthrombi in the subcutaneous vessels on pathological examination of the skin specimen and by the improvement of the platelet counts under continuous heparin administration. Based on laboratory and clinical findings, SLE and ANCA-associated vessels were also ruled out. Although thrombocytopenia caused by drugs other than IVIg was suspected, the thrombocytopenia resolved spontaneously without any drug change except for IVIg. Based on these results, the patient was diagnosed with thrombocytopenia due to IVIg. Furthermore, the Naranjo Adverse Drug Reaction Probability Scale [9] evaluated the likelihood of a connection between IVIg and thrombocytopenia. The score was 6 points, an indicating that the association was probable. The thrombocytopenia occurred rapidly during IVIg administration for TEN, and the platelet count increased after approximately 10 days. Generally, drug-induced immune thrombocytopenia occurs approximately 14 days after drug initiation [10]. In the present case, thrombocytopenia was observed on the third day of IVIg administration, which is an early onset compared with the general course of drug-induced immune thrombocytopenia; however, this is similar to previous reports of thrombocytopenia due to IVIg [5, 11]. The platelet count in patients with drug-induced thrombocytopenia typically improves within approximately 8 days after discontinuation of the causative drug, and 70% of patients show improvement within 2 weeks [12]. In the present case, thrombocytopenia improved within approximately 10 days, which was similar to previously reported cases of IVIg-induced thrombocytopenia, which showed an improvement within approximately 10–14 days [11, 12]. Therefore, IVIg-induced thrombocytopenia should be suspected when thrombocytopenia occurs during IVIg administration and resolves spontaneously within 10 to 14 days. The quick onset of this condition may occur through a mechanism unrelated to an antigen–antibody reaction, distinguishing it from typical drug-induced immune thrombocytopenia. IVIg reacted more strongly with the platelet surface glycoproteins GPIIbIIIa and GPIaIIa in hypogammaglobulinemic patients with IVIg-induced thrombocytopenia than in healthy individuals in vitro, resulting in the formation of IgG–PLT complexes in a dose-dependent manner [13]. While we assume that IgG–PLT complexes are removed by the intra-retinal system, leading to thrombocytopenia [13]; in the present case, PAIgG was also detected, indicating this mechanism may have been relevant. Autoimmunity and inflammatory status can affect the incidence of hematological adverse events caused by IVIg via macrophage activation [14, 15], and TEN is associated with similar risks. Additionally, thrombocytopenia with freeze-dried polyethylene glycol-treated human normal immunoglobulin used in the present case has been reported [5, 11, 16], suggesting that the product may be associated with the development of thrombocytopenia. Thus, the mechanism and factors related to IVIg-induced thrombocytopenia are unclear and further studies are needed.

This case report has notable limitations. First, although numerous diseases that can lead to thrombocytopenia were excluded, some were not. For example, ADAMTS13 activity was not assessed, leaving the possibility of thrombotic thrombocytopenic purpura undetermined. Nevertheless, as thrombocytopenia resolved independently, the likelihood of new diseases, such as thrombotic thrombocytopenic purpura is low. Second, although it is assumed that the development of IgG–PLT complexes relates to IVIg-induced thrombocytopenia, the number of reported case IgG–PLT complexes is few, making its role uncertain. Lastly, this is a single case report, necessitating further studies to explore the connection between IVIg and thrombocytopenia, including basic research and case series.

In conclusion, TEN is associated with a high rate of organ failure and infection, and its mortality rate is extremely high, particularly in patients with renal failure. Therefore, as observed in the present case, thrombocytopenia during TEN treatments may lead to severe outcomes due to hemorrhagic complications. Although cases of IVIg-induced thrombocytopenia are rare, the risk of thrombocytopenia should be recognized and the platelet count should be carefully monitored when IVIg is administered for TEN.

## Data Availability

Not applicable.

## Abbreviations

TEN: Toxic epidermal necrolysis
IVIg: Intravenous immunoglobulin
DIC: Disseminated intravascular coagulation
APS: Antiphospholipid antibody syndrome
HIT: Heparin-induced thrombocytopenia
PAIgG: Platelet-associated IgG
